# Analysis of the Preventive Effect of *Lonicera caerulea* Pomace and Its Isolated Components on Colitis in Mice Based on Gut Microbiota and Serum Metabolomics

**DOI:** 10.3390/antiox13121478

**Published:** 2024-11-30

**Authors:** Zinuo Zhou, Xinwen Huang, Baixi Zhang

**Affiliations:** 1 School of Food Science and Technology, Jiangnan University, Wuxi 214122, China; 6230111160@stu.jiangnan.edu.cn (Z.Z.).; 2 National Engineering Research Center for Functional Food, Jiangnan University, Wuxi 214122, China

**Keywords:** *Lonicera caerulea*, polyphenol, anti-inflammatory, gut microbiota, metabolomics

## Abstract

Inflammatory bowel disease (IBD), including relapsing-remitting ulcerative colitis and Crohn’s disease, is a non-specific chronic intestinal inflammatory disease. *Lonicera caerulea*, which is rich in polyphenolic compounds, has been shown to exert antioxidative and anti-inflammatory effects. The research evaluates the dietary impacts of *Lonicera caerulea* pomace, its polyphenol-rich extract, and fiber-rich residue on colitis symptoms. Colitis was induced with 2.5% DSS (dextran sulfate sodium) aqueous solution after continuous feeding of customized *Lonicera caerulea* feed for 2.5 weeks. The results indicate that the intake of the polyphenol-rich extract has an effect in preventing colitis in mice, but the effect is less than that by the pomace itself, and the fiber residue alone does not prevent the condition when ingested. The pomace and polyphenol-rich extract have a positive regulatory effect on the gut microbiota of mice with colitis, and the intake of *Lonicera caerulea* pomace significantly restores 15 metabolites in mice with colitis, significantly improving five metabolic pathways, including steroid biosynthesis, with the regulation of metabolites and metabolic pathways being significantly correlated with the gut microbiota.

## 1. Introduction

Crohn’s disease and ulcerative colitis (UC) are two types of inflammatory bowel disease (IBD) [1], both of which are chronic idiopathic diseases caused by gastrointestinal inflammation. Their clinical manifestations are diarrhea, hematochezia, abdominal pain, weight loss, etc. [2]. The inflammation in UC is characterized by colonic tissue damage, inflammatory cell infiltration, and overproduction of proinflammatory cytokines in the gastrointestinal tract [3]. The specific pathogenesis of UC is still unclear, but it is generally believed that its pathogenesis includes genetic susceptibility, mucosal barrier dysregulation, intestinal flora imbalance, and other environmental factors [2,4]. In recent years, more and more attention has been paid to the impact of the intestinal flora on colitis. Many studies have shown that the intestinal flora of patients with UC shows reduced diversity, changes in the abundance of specific groups, and changes in biological functions [5,6,7,8]. The intestinal flora plays an important role in the occurrence and treatment of UC [9]. In addition, compared with traditional therapies with serious side effects, dietary therapy has become an effective and simple way to shape the gut microbiota [10,11,12].

*Lonicera caerulea* is a plant of the family *Caprifoliaceae*, belonging to the genus Lonicera [13]. Its fruit ripens early and has high cold and frost resistance. It can survive in low temperatures of −50 °C in the Greater Khingan Mountains. The plant composition of *Lonicera caerulea* varies depending on different geographical locations and varieties and is related to the harvest date [14]. This fruit has a sour to sweet taste, with high fructose and glucose levels, and a small amount of sucrose [15]. Due to the rich content of phenolic and organic acids in *Lonicera caerulea*, it is usually accompanied by a slightly sour taste when consumed, but this also gives it strong antioxidant and anti-inflammatory properties [16]. LCP (*Lonicera caerulea* pomace) is a byproduct of juice processing, rich in dietary fiber and polyphenols [17]. Among all phenolic compounds found in LCP, anthocyanins account for the largest proportion, ranging from 36% to 51%, with cyanidin-3-O-glucoside (C3G) being the most important [18,19,20]. The regular consumption of fruits and vegetables rich in phytochemicals has been associated with an improvement in the symptoms of UC and other IBD [5,21,22]. However, in phytochemicals, polyphenol conjugates cannot be digested in the small intestine, but they can be released in the large intestine through microbial fermentation and are broken down by the gut microbiota into small molecule metabolites, playing a role there [23]. There are studies reporting that water-soluble dietary fiber protects the breakdown of C3G in the gastrointestinal tract [23,24,25]. Loo et al. [23,26] combined polyphenol-rich sugarcane extract with fiber and found that it could increase the availability of phenolic compounds in the colon [26]. Dietary fiber limits the bioavailability of phenolic substances in the small intestine, but they act as fermentation substrates for the gut microbiota and regulate it to a more favorable state [27]. However, existing research on the beneficial effects of LCP on intestinal health is limited, and the effects of LCP extract on colitis have not been reported.

In this study, we established a DSS (dextran sulfate sodium)-induced colitis model using C57BL/6J mice to investigate the preventive effects of LCP, polyphenol extract, and fiber residue on colitis. We evaluated the effects of these components on clinical symptoms, colon characteristics, tissue integrity, inflammatory markers, and tight junction protein expression in mice. In addition, we used 16SrRNA sequencing to analyze the gut microbiome and conducted serum metabolomic studies to elucidate the potential mechanisms by which LCP and its derived components affect colitis.

## 2. Materials and Methods

### 2.1. Chemical and Reagents

The Plant Total Phenol (TP) Content Assay Kit was obtained from Sangon Biotech Co., Ltd. (Shanghai, China). DSS (molecular weight = 36,000–50,000 Da) was obtained from MP Biomedicals (Irvine, CA, USA). The commercial kit for myeloperoxidase (MPO) was obtained from Nanjing Jiancheng Bioengineering Institute (Nanjing, China). The ELISA kit for the determination of tumor necrosis factor-α (TNF-α) was obtained from Shanghai Enzymelinked Biological Technology Co., Ltd. (Shanghai, China). The other chemicals were commercially accessible and analytical grade.

### 2.2. LCP Preparation and Supplemental Dose Evidence

*L. caerulea* berries were obtained from FengRan Agricultural Group Company (Jiamusi City, China), the variety of which was ‘Beilei’. A juice extractor (VP21E) from Slovenia was used for pressing the fruit mass without adding water (the juice extraction efficiency was 80%). The pomace was collected and stored at −16 °C for further processing.

The AIN-93M diets were supplemented with 5% and 10% LCP by weight, manufactured by Trophic Animal Feed High-Tech Co., Ltd. (Nantong, China). The diet formula was adjusted to keep the calorie composition of the LCP diet consistent with that of the control diet. The supplemental dose of LCP was based on the amount of grape pomace and other pomaces with a similar composition as LCP added to the diet in colitis alleviation experiments [8,28,29]. The dose of 5% LCP supplement was 50 g/kg of the diet. The average dietary intake of a mouse weighing 22 g was 3 g/mouse/day, which equaled 150 mg LCP per day (6.82 g LCP/day/kg bw). A 6.82 LCP/day/kg bw for a mouse converts to 33.17 g of LCP dietary intake per day for a 60 kg human using the body surface area normalization method [30].

### 2.3. Preparation and Nutrient Composition Determination of LCP and Its Separated Components

Remove the *Lonicera caerulea pomace* from the −80 °C refrigerator, freeze-dry it, grind it into powder using a grinder, and sieve it through a 100-mesh screen to obtain *Lonicera caerulea* pomace powder. To separate the two components rich in polyphenols and dietary fiber in the *Lonicera caerulea* pomace, we referenced previous polyphenol ethanol extraction methods [8,13,15] and made modifications. Take 1 g of *Lonicera caerulea* pomace powder, add 40 mL of extraction solvent (ethanol:water = 1:1, volume ratio, adjusted to pH 2 with hydrochloric acid), stir to evenly distribute the powder in the extraction solvent, and extract for 60 min at room temperature (25 °C) with magnetic stirring. Afterward, filter the residue and filtrate separately, extract the residue a second time under the same conditions, and filter again. Combine the two extraction filtrates, and use a rotary evaporator to concentrate them to 1/10 of the original volume at 39 °C under vacuum to obtain the polyphenol-rich alcohol-water extract (PEE) of the fruit residue. Collect the residue, wash it with pure water 2–3 times, freeze-dry to remove the water, grind it into powder using a grinder to obtain the dietary fiber-rich alcohol-water extract residue (fiber residue).

Ash and crude protein were analyzed according to the Association of Official Analytical Chemists [31]. Total and insoluble dietary fiber were determined by the enzymatic-gravimetric method [32].Soluble sugars were extracted using 80% ethyl alcohol and spectrophotometrically quantified using the phenol-H2SO4 assay [33].

### 2.4. Qualitative and Quantitative Analysis of Polyphenols

#### 2.4.1. Sample Pretreatment

Pipette 100 μL of alcoholic aqueous extract of pomace (PEE) into a centrifuge tube, add 300 μL of chromatographic-grade methanol, vortex and mix for 30 s, sonicate for 10 min (in an ice-water bath), let stand at −40 °C for 1 h, and centrifuge at 4 °C and 12,000× *g* rpm for 15 min. Take the supernatant, filter it through a 0.45 μm organic membrane filter and then proceed to instrument detection.

#### 2.4.2. Pretreatment of Standard Samples

Accurately weigh 5 mg of standard substance into a 25 mL volumetric flask, add chromatographically pure methanol to dissolve and dilute to volume, obtaining a 200 ppm standard substance stock solution. Dilute the stock solution with methanol to obtain solutions of 2, 10, 50, 100, and 500 ppb, which are used to prepare the standard curve.

#### 2.4.3. Qualitative Analysis Conditions for Polyphenols Chromatographic Conditions

LC-MS/MS analyses were performed using an UHPLC system (Vanquish, Thermo Fisher Scientific, Waltham, MA, USA) with a UPLC HSS T3 column (2.1 mm × 100 mm, 1.8 μm) coupled to an Orbitrap Exploris 120 mass spectrometer (Orbitrap MS, Thermo Fisher Scientific, Waltham, MA, USA). The mobile phase consisted of 5 mmol/L ammonium acetate and 5 mmol/L acetic acid in water (A) and acetonitrile (B). The auto-sampler temperature was 4 °C, and the injection volume was 2 μL. The Orbitrap Exploris 120 mass spectrometer was used for its ability to acquire MS/MS spectra in information-dependent acquisition (IDA) mode under the control of the acquisition 4.4 software (Xcalibur, Thermo Fisher Scientific, Waltham, MA, USA). In this mode, the acquisition software continuously evaluated the full-scan MS spectrum. The ESI source conditions were set as follows: sheath gas flow rate of 50 Arb; Aux gas flow rate of 15 Arb; capillary temperature of 320 °C; full MS resolution of 60,000; MS/MS resolution of 15,000; collision energies of 10/30/60 in NCE mode; and spray voltage of 3.8 kV (positive) or −3.4 kV (negative), respectively.

#### 2.4.4. Chromatographic Conditions for Quantitative Analysis of Polyphenols

A Waters BEH C18 (2.1 mm × 100 mm, 1.7 μm) liquid chromatography column was used, with mobile phase A consisting of 0.1% aqueous formic acid and mobile phase B consisting of acetonitrile. The elution gradient was as follows: 0~1 min, 2% B; 1~10 min, 2%~98% B; 10~12 min, 98% B; 12~12.1 min, 98%~2% B; 12.1~15 min, 2% B. The flow rate was 0.30 mL/min, the column temperature was 40 °C, and the injection volume was 1 μL. The mass spectrometry conditions referred to the qualitative analysis method described in Section 2.4.3.

### 2.5. Animal Experimental Design

The animal experiment was reviewed and authorized by Jiangnan University Experimental Animal Management and Animal Welfare Ethics Committee (JN. No. 20220615c0700707 [208]). The experimental animals were purchased from GemPharmatech (Beijing, China) and housed in a specific pathogen-free environment at the Laboratory Animal Center of Jiangnan University (license no. SYXK (Su) 2021-0056, Wuxi, China) in a constant temperature (20–26 °C), humidity (40–70%), and light/dark cycle (12 h/12 h) environment. The experimental schemes were as follows. All mice received sterile water and standard diet (AIN-93M) to acclimatize for one week, and then were randomly divided into 5 groups (*n* = 8): (1) Control group, (2) DSS group, (3) DSS + *L. caerulea* pomace (LCP) group (LCP was added to the standard diet at 10% by weight and the diet formulation was adjusted to achieve the same caloric ratio as the standard diet), (4) DSS + polyphenol-rich ethanol extract of LCP (PEE) group (the ethanol extract obtained from the corresponding amount of pomace from the DSS + LCP diet was separated and added to the diet), and (5) DSS + fiber-rich ethanol extract residue of LCP (residue) group (the amount of residue in the diet was equivalent to the residue obtained after extraction of the same amount of LCP used in the DSS + LCP diet). All customized diets were formulated and adjusted to achieve the same caloric ratio as the standard diet (Table 1). The standard diet and the customized diets were provided by Nantong TROPHY Feed Technology Co., Ltd. To induce colitis in all groups except the control group, sterile water in drinking bottles was substituted with 2.5% (*w*/*v*) DSS aqueous solution on days 18 to 25 [34]. Mice were sacrificed on day 25 of the experiment after a 12 h fast and mouse feces were collected. Before collecting the colon into sterile tubes, the colon length was measured. Following the acquisition of 1 cm tissues for hematoxylin and eosin (H&E) staining, colon tissues were quickly frozen in liquid nitrogen for further examination.

### 2.6. Myeloperoxidase (MPO) and Enzyme-Linked Immunosorbent Assay (ELISA)

Colon tissue was accurately weighed, and 5% tissue homogenate was prepared by adding precooled PBS (0.01 M, pH = 7.4) at a ratio of 1:19 (*w*/*v*). The tissue homogenate was subsequently homogenized with distilled water and chromogenic solution, followed by incubation in a water bath at 37 °C for 30 min. The reaction was terminated with a 10 min bath at 60 °C after stop solution was added. Absorbance was measured immediately, using a 460 nm wavelength and a 1 cm optical path. A unit of MPO activity is described as the amount of 1 mol H_2_O_2_ degraded at 37 °C, with the value represented in units per gram (U/g). According to the protocol of the ELISA kit, 50 μL of stop solution was added to the serum solution, and the absorbance at a 450 nm wavelength was measured immediately to determine the concentration of TNF-α in the mouse serum.

### 2.7. Immunohistochemical Analysis

Paraffin sections were dewaxed in water, and antigen repair was performed in citric acid antigen repair buffer in a microwave oven. The sections were naturally cooled and washed 3 times with phosphate-buffered solution for 5 min each. The sections were placed in 3% hydrogen peroxide, incubated for 25 min at room temperature protected from light, and washed 3 times for 5 min each. Next, 3% bovine serum albumin was added dropwise in the histochemical circle to cover the tissue evenly, and the sections were incubated at room temperature for 30 min and gently shaken off. The sections were incubated overnight at 4 °C in a wet box, washed 3 times for 5 min each, and the sections were slightly shaken dry, covered with secondary antibody in the circle, incubated for 50 min at room temperature, and washed 3 times for 5 min each. The sections were slightly shaken dry and covered with freshly prepared DAB chromogenic solution in the circle, the color development time was controlled under the microscope (positive color was brownish yellow), and the sections were rinsed with tap water to terminate color development. Hematoxylin was used to re-stain the nuclei, the sections were sealed by dehydration, microscopic examination was performed, and images were collected for analysis. The images were analyzed using the software Image J 1.53. for data-based analysis.

### 2.8. Serum Metabolomics Analysis

Take 100 μL of serum into a centrifuge tube, add 700 μL of extractant (methanol:acetonitrile:water = 4:2:1, *v*/*v*), shake for 1 min, and place in a refrigerator at −20 °C for 2 h. Remove from the tube, centrifuge at 25,000× *g* for 15 min at 4 °C, transfer 600 μL of supernatant to a new tube, lyophilize, add 180 μL of methanol:pure water (1:1, *v*/*v*), shake for 10 min until re-solubilization, centrifuge again at 4 °C at 25,000× *g* for 15 min, and transfer the supernatant to a new tube. After lyophilization, add 180 μL of methanol:pure water (1:1, *v*/*v*), shake for 10 min until re-solubilization, centrifuge again at 4 °C for 15 min at 25,000× *g*, and remove the supernatant to a new tube. A Waters UPLC I-class plus (Waters, Milford, MA, USA) tandem Q Exactive high-resolution mass spectrometer (Thermo Fisher Scientific, Waltham, MA, USA) was used to separate and detect the metabolites.

Chromatographic conditions: A Waters BEH C18 (2.1 mm × 100 mm, 1.7 μm) liquid chromatographic column was used. Positive ionization mode mobile phase A was aqueous solution containing 0.1% formic acid and phase B was methanol containing 0.1% formic acid; negative ionization mode mobile phase A was aqueous solution containing 10 mM ammonium formate and phase B was 95% methanol containing 10 mM ammonium formate. The elution gradient was 0~1 min, 2% B; 1~9 min, 2%~98% B; 9~12 min, 98% B; 12~12.1 min, 98%~2% B; 12.1~15 min, 2% B. The flow rate was 0.35 mL/min, the column temperature was 45 °C, and the injection volume was 5 μL.

Mass spectrometry conditions: Mass spectrometry scan *m*/*z* range 70~1050. ESI source conditions were set as follows: sheath gas flow rate of 40 Arb; auxiliary gas flow rate of 10 Arb; capillary temperature of 320 °C; primary resolution of 70,000; secondary resolution of 17,500; collision energies of 20, 40, and 60 eV; and spray voltage of 3.8 kV (positive ions) or −3.2 kV (negative ions), respectively.

The mass spectrometry data were imported into Compound Discoverer 3.3 software (Thermo Fisher Scientific, Waltham, MA, USA) combined with BMDB (UW Metabolome Database), the mzCloud database, and the ChemSpider online database for mass spectrometry data analysis.

### 2.9. Fecal Microbial Gene Sequencing and Analysis

After 7 days of DSS administration, fecal samples were collected to extract the genomic DNA. There were five groups, each with 8 independent samples. The PCR reaction system was configured using 30 ng of qualified genomic DNA and V3-V4 region primers. The primers were 341F (5′-ACTCCTACGGGAGGCAGCAG-3′) and 806R (5′-GGACTACHVGGGTWTCTAAT-3′). The PCR products were purified, dissolved using Agencourt AMPure XP obtained from Beckman Coulter, Inc. (Pasadena, CA, USA), and tagged to construct libraries. The qualified libraries were sequenced on a HiSeq platform from BGI Genomics (Shenzhen, China). Cutadapt (v.2.6) and readfq (v1.0) were used to filter the raw data. Fast Length Adjustment of Short reads (v1.2.11) was used for generating consensus sequences. The denoising method of the divisive amplicon denoising algorithm was used to obtain amplicon sequence variants, after which the feature table was obtained. The representative OTU sequences were compared with the database using RDP classifier (v2.2) for taxonomic annotation. Diversity analysis, function prediction, and other data analysis were based on OTU and taxonomic annotation. Here, we selected the samples of the P10 group for 16S rRNA gene sequencing and annotated them as pomace group (P) in the results section.

### 2.10. Statistical Analysis

All data are expressed as the mean ± standard deviation (SD), using one-way analysis of variance (ANOVA), post-hoc LSD test, and the Tamhane test. Significant differences were assessed using SPSS (Version 25), the significance level of differences is indicated by the symbols * *p* < 0.05, ** *p* < 0.01, and *** *p* < 0.001, and “ns” indicates no significant difference. The data were graphically visualized using GraphPad Prism 8.0.

## 3. Results

### 3.1. Compositional Analysis of Separated Fractions of LCP

The nutrient compositions of the polyphenol-rich ethanol extract (PEE) and the fiber-rich ethanol extract residue of LCP were determined and the results are shown in Table 2. It was discovered that dietary fiber accounted for 65.20% of the residue of the ethanolic extract and was the major component.

Two hundred substances were identified in PEE by HPLC-MS, 19 polyphenols were selected for further analysis by mass spectrometry score and literature search screening of *Lonicera caerulea* components to quantify the polyphenolic chemicals in PEE, and the results are shown in Table 3. The substances with the highest contents in LCP were cyanidin-3-o-glucoside and cyanidin-3-O-galactoside, with 388.31 ± 8.93 mg/100 g pomace and 231.35 ± 2.31 mg/100 g pomace, respectively. In addition, among the detected chemical substances, many had not been quantitatively reported in *Lonicera caerulea*, such as astragalin, naringenin, daidzin, neodiosmin, cyanidin-3-O-galactoside, aromadendrin, amentoflavone, 1,5-dicaffeoylquinic acid, and 7-hydroxycoumarin. Due to its isomer with C3G (cyanidin-3-O-glucoside), the quantification of cyanidin-3-O-galactoside may have been overlooked in previous studies. The detected content was 231.35 ± 2.31 mg/100 g in *Lonicera caerulea* pomace, which was of the same order of magnitude as the content of C3G. In addition, among quantitative compounds, there are many reports on the inhibition of colitis. The main pathways for inhibiting colitis by astragaloside, daidzin, chlorogenic acid, and naringenin include anti-inflammation, antioxidation, and regulation of intestinal flora [7,13,14,26]. These studies help to explain the subsequent alleviating effects of fruit pomace and its alcohol-water extract on colitis.

### 3.2. The Ameliorative Effects of LCP and Its Fractions on Colitis Symptoms

**Figure 1:**
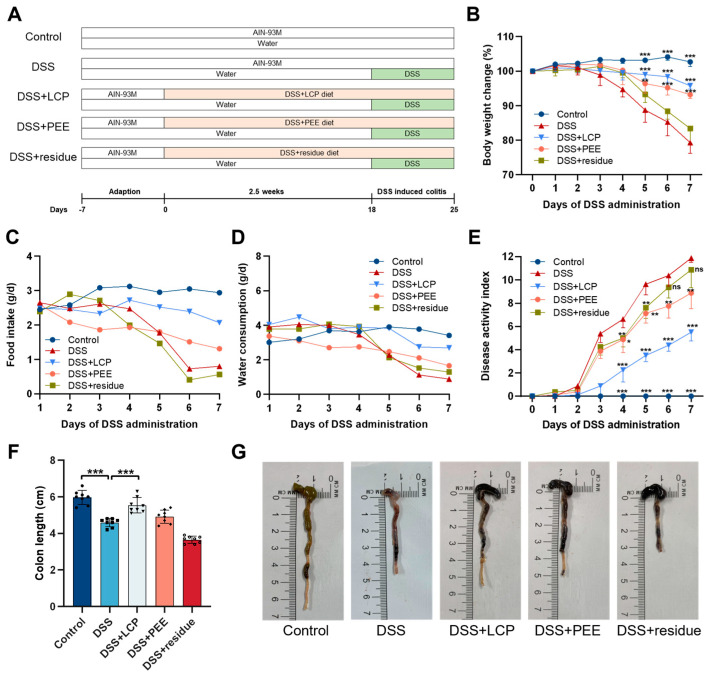
Experimental scheme and effects of LCP and its fractions on DSS-induced mice colitis symptoms. (**A**) Experimental scheme. (**B**) Body weight change. (**C**) Food intake. (**D**) Water consumption. (**E**) Disease activity index. (**F**) Colon length. (**G**) Representative images of the mouse colon. Data are presented as mean ± SD (*n* = 8). * *p* < 0.05, ** *p* < 0.01, *** *p* < 0.001, and “*” marked in the B and E diagrams indicate significant differences compared with the DSS group. “ns” denotes comparisons with no significance. One-way ANOVA with post-hoc Tukey test.

From the 4th day of DSS administration, the weights of the colitis mice in the DSS group started to decrease significantly (*p* < 0.001), and the rates of weight change in the DSS + LCP and DSS + PEE groups were always significantly different from that of the DSS group (*p* < 0.001), with the rate of decrease being slower, while there was no significant difference between the DSS + residue group and the DSS group (Figure 1B). The food intake and water consumption of the DSS + LCP group were close to those of the control group, those of the DSS + PEE group were slightly higher than those of the DSS group, and those of the DSS + residue group were close to those of the DSS group (Figure 1C,D). The disease activity index (DAI) is an important indicator of the severity of intestinal inflammation and is evaluated based on weight loss, stool consistency, and blood in the stool [34,35]. The DAI of mice in the DSS group showed a significant increase (*p* < 0.01) from the 4th day of DSS administration and they began to show loose stools and positive occult blood, and the DAI of all groups displayed an increasing trend over time. On the 7th day, we found that there were significant differences between the DSS + LCP and DSS + PEE groups compared with the DSS group (*p* < 0.01), with more significant differences in the DSS + LCP group (*p* < 0.001) and no significant differences between the DSS + residue group and the DSS group (Figure 1E). Compared with the DSS group (4.59 ± 0.22 cm), LCP significantly increased the colon length of colitis mice to 5.54 ± 0.42 cm (*p* < 0.001), which recovered to the level of the control group (5.96 ± 0.38 cm) (Figure 1F). As observed from the morphology of the colon, the state of the DSS + LCP group was close to that of the control group(only the color of the contents was dark), and the traits of the DSS + PEE group were similar to those of the DSS + LCP group (Figure 1G). This indicated that LCP intake significantly inhibited colon shortening and restored the content and morphology of the colon, while PEE intake maintained the morphology similar to that in the DSS + LCP group, and the restoration of the colon traits in the DSS + residue group was not effective.

### 3.3. Effects of LCP and Its Fractions on Colon Tissue Damage and the Inflammatory Response Associated with Colitis

**Figure 2:**
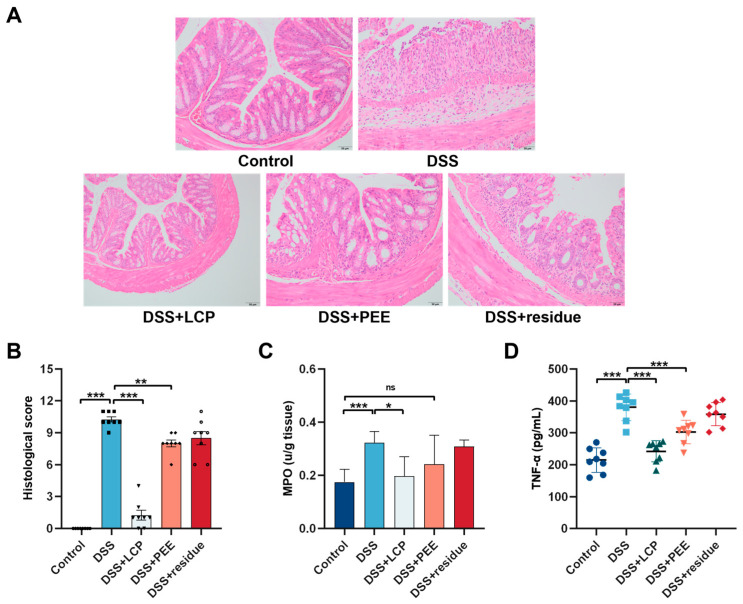
Effects of LCP and its fractions on colon histology and the inflammatory response. (**A**) Histological examination, scale bars, 20 μm. (**B**) Histological score. (**C**) MPO activity in colon tissues. (**D**) Concentration of TNF-α in serum. Data are presented as mean ± SD (*n* = 8). * *p* < 0.05, ** *p* < 0.01, *** *p* < 0.001, and “ns” denotes comparisons with no significance. One-way ANOVA with post-hoc Tukey test.

The DSS group showed depletion of goblet cells and loss of superficial epithelial cells, large inflammatory infiltrates in the mucosa and submucosa, and edema between the mucosal and muscular layers of the intestine. Compared with the DSS group, the DSS + LCP group showed significantly less pathological damage and the morphology approached that of the control group. The DSS + PEE group showed moderately severe inflammation, 1/2 of the goblet cells were damaged, edema was slightly improved, and there were still some inflammatory cell infiltrates. The DSS + residue group showed the loss of most of the crypt structure, the superficial epithelial cells were broken, and there were some inflammatory cell infiltrates (Figure 2A). The histopathological scores showed that, compared with the DSS group, the DSS + LCP group showed an 87.80% decrease in score and the DSS + PEE group showed a 21.95% decrease in score, both with significant differences (*p* < 0.01), and the DSS + residue group showed a 17.07% decrease in score, with no significant difference (*p* > 0.05) (Figure 2B).

MPO activity and TNF-α are indicators of characterized inflammation. Compared with the control group, DSS significantly increased MPO activity and LCP intake significantly alleviated the DSS-induced increase in MPO activity (*p* < 0.05), with no significant differences between the DSS + PEE, DSS + residue, and DSS groups (Figure 2C). We found that the DSS + LCP and DSS + PEE groups showed markedly attenuated DSS-induced increases in serum TNF-α (*p* < 0.001), with no significant differences between the DSS + residue and DSS groups (Figure 2D).

### 3.4. Effect of LCP and Its Fractions on Colonic Tight Junction Proteins in Colitis Mice

**Figure 3:**
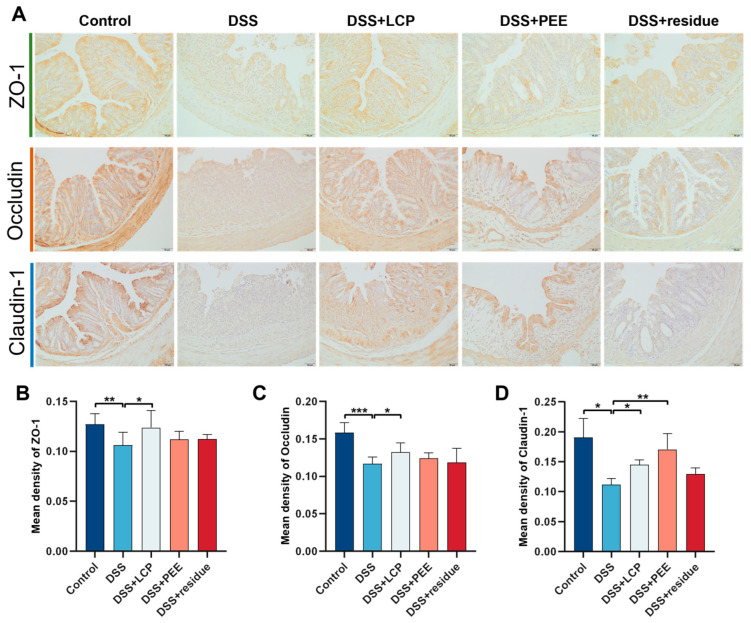
Effect of LCP and its fractions on colonic tight junction proteins in colitis mice. (**A**) Immunohistochemical staining images, scale bars, 50 μm. (**B**–**D**) Mean optical densities of ZO-1, Occludin, and Claudin-1 in colon tissues. Data are presented as mean ± SD (*n* = 8). * *p* < 0.05, ** *p* < 0.01, *** *p* < 0.001, and “ns” denotes comparisons with no significance. One-way ANOVA with post-hoc Tukey test.

We analyzed the tight junction proteins ZO-1, Occludin, and Claudin-1 in colon tissues by immunohistochemical staining techniques to evaluate and compare the protective effect of LCP and each of its isolated fractions on the mechanical barrier of the colon. Compared with the DSS group, the mean optical densities of ZO-1, Occludin, and Claudin-1 in the DSS + LCP group markedly increased by 16.30%, 13.18%, and 29.47% (*p* < 0.05), respectively, which were close to the control levels; the DSS + PEE group significantly increased the mean optical density of Claudin-1 by 52.20% (*p* < 0.01) and slightly increased the mean optical densities of ZO-1 and Occludin (*p* > 0.05); and there was no significant difference between the DSS + residue group and the DSS group (Figure 3).

### 3.5. Modulation of LCP and Its Fractions on the Gut Microbiota

**Figure 4:**
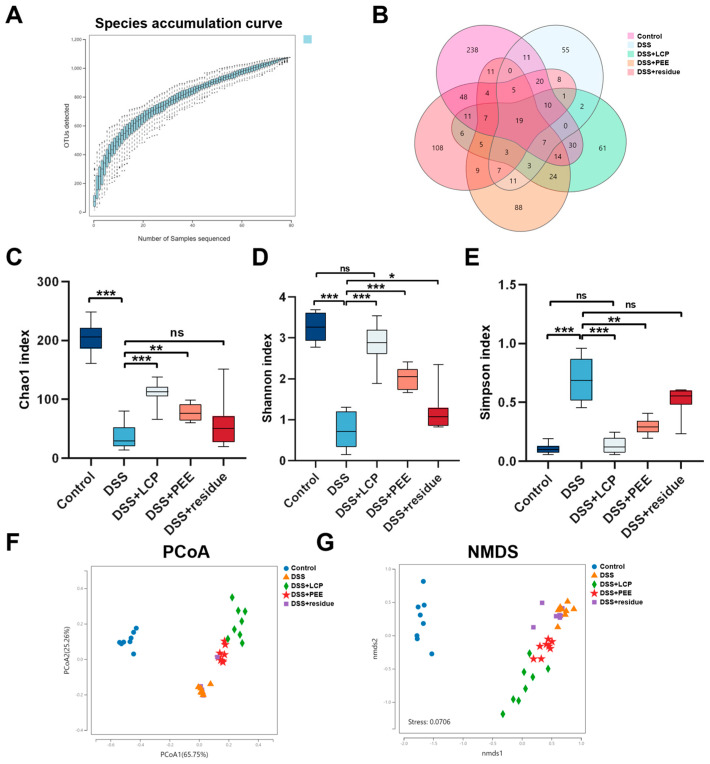
Changes in abundance and diversity of the gut microbiota. (**A**) Species accumulation curve. (**B**) Venn diagram. (**C**) Chao 1 index. (**D**) Shannon index. (**E**) Simpson index. (**F**) PCoA plot of structural changes in the gut microbiota. (**G**) NMDS plot of structural changes in the gut microbiota. Data are presented as mean ± SD (*n* = 8). * *p* < 0.05, ** *p* < 0.01, *** *p* < 0.001, and “ns” denotes comparisons with no significance. One-way ANOVA with post-hoc Tukey test.

It is well known that the dynamic balance of the gut microbiota is influenced by the diet [35,36,37] and that the gut microbiota plays an indispensable role in the development of colitis [36,37]. To illustrate the effect of LCP and its fractions on the gut microbiota of colitis mice, we performed a microbiological analysis of mouse feces by 16s rRNA sequencing. According to the species accumulation curves (Figure 4A), there was no significant increase in species as the number of samples increased, indicating that the sample size was sufficient to reflect the species richness. The Venn diagram demonstrates the overlap of OTUs between samples, with 238, 55, 61, 88, and 108 unique microorganisms in the Control, DSS, DSS + LCP, DSS + PEE and DSS + residue groups, respectively (Figure 4B). The DSS + LCP and DSS + PEE groups showed significantly increased Chao1 indexes and the reduction in species abundance due to colitis was alleviated (Figure 4C). Compared with the DSS group, the DSS + LCP and DSS + PEE groups showed significantly improved Shannon indexes and the Simpson indexes were decreased, indicating the restoration of gut microbiota diversity, with no significant difference between the DSS + LCP group and the Control group, and the DSS + residue group only showed an improved Shannon index (Figure 4D,E).

Based on principal component analysis (PCoA), it was found that the samples from the control, DSS, DSS + LCP, and DSS + PEE groups clustered distinctly and were significantly separated between the groups, indicating that there were significant differences in the colon structure of these four groups (Figure 4F). Analysis using a combination of non-metric multidimensional scaling (NMDS) and Anosim revealed that there were significant differences and groupings among the four groups of samples from the control, DSS, DSS + LCP, and DSS + PEE groups, while the groupings of the samples from the DSS + residue and DSS groups were not significant (Figure 4G). This suggested that dietary LCP and PEE intake had a significant effect on the change in community composition due to colitis, while the residue had no significant effect and was not meaningfully grouped with the DSS group. Given that the DSS + residue group did not show significant improvements in any of the previous colitis indicators, the DSS + residue group was excluded from subsequent analyses.

**Figure 5:**
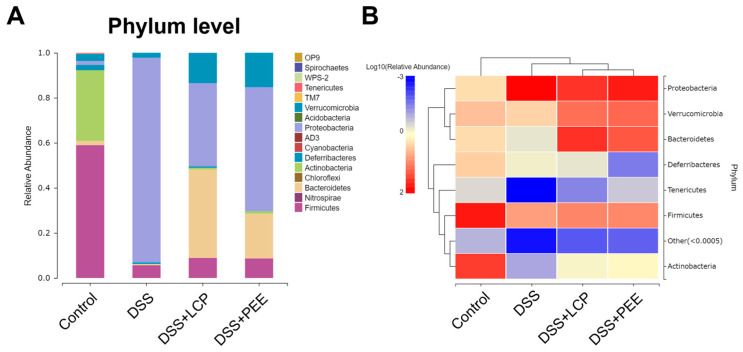
Phylum-level analysis of the species composition of the gut microbiota. (**A**) Phylum-level histogram. (**B**) Heat map. Data are presented as mean ± SD (*n* = 8).

At the phylum level, differences in the species composition of the gut microbiota were explored (Figure 5). Compared with the DSS group, the abundance of *Proteobacteria* was significantly reduced by 59.26% in the DSS + LCP group, and the abundances of *Bacteroidetes*, *Tenericutes*, *Actinobacteria*, and *Verrucomicrobia* were significantly increased, 76.6, 52.3, 8.7, and 6.3 times higher than those of the DSS group, respectively; the abundance of *Proteobacteria* was significantly reduced by 39.39% in the DSS + PEE group, and the abundances of *Bacteroidetes*, *Actinobacteria*, and *Verrucomicrobia* were significantly increased, 38.7, 11.8, and 7.1 times higher than those of the DSS group.

**Figure 6:**
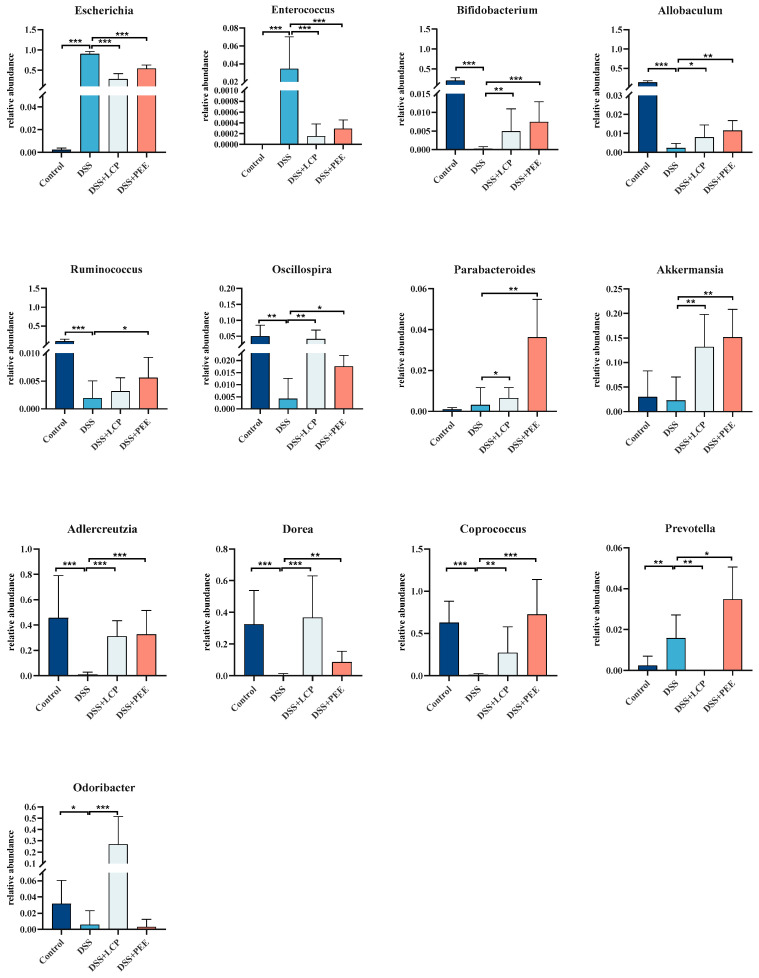
Species with significant changes at the genus level. Data are presented as mean ± SD (*n* = 8). * *p* < 0.05, ** *p* < 0.01, *** *p* < 0.001, and “ns” denotes comparisons with no significance. One-way ANOVA with post-hoc Tukey test.

Genus-level analysis of each species showed that, compared to the DSS group, the abundances of *Escherichia* and *Enterococcus* were significantly lower in the DSS + LCP and DSS + PEE groups, and the abundances of *Bifidobacterium*, *Allobaculum*, *Oscillospira*, *Parabacteroides*, *Akkermansia*, *Adlercreutzia*, and *Coprococcus* were significantly higher (*p* < 0.05). In addition, there was a trend of increased abundance of *Ruminococcus* in the DSS + LCP and DSS + PEE groups compared to the DSS group, but it was not significant (*p* > 0.05) (Figure 6). This suggested that colitis led to a decrease in the abundance of beneficial bacteria and an increase in the abundance of pathogenic bacteria in the intestine, while the intake of dietary LCP and PEE enriched the probiotic bacteria (*Bifidobacterium*, *Allobaculum*, *Oscillospira*, *Akkermansia*, *Adlercreutzia*, *Coprococcus*, and *Odoribacter*), decreased the abundance of harmful bacteria (*Escherichia* and *Enterococcus*), and improved the species distribution of the gut microbiota.

**Figure 7:**
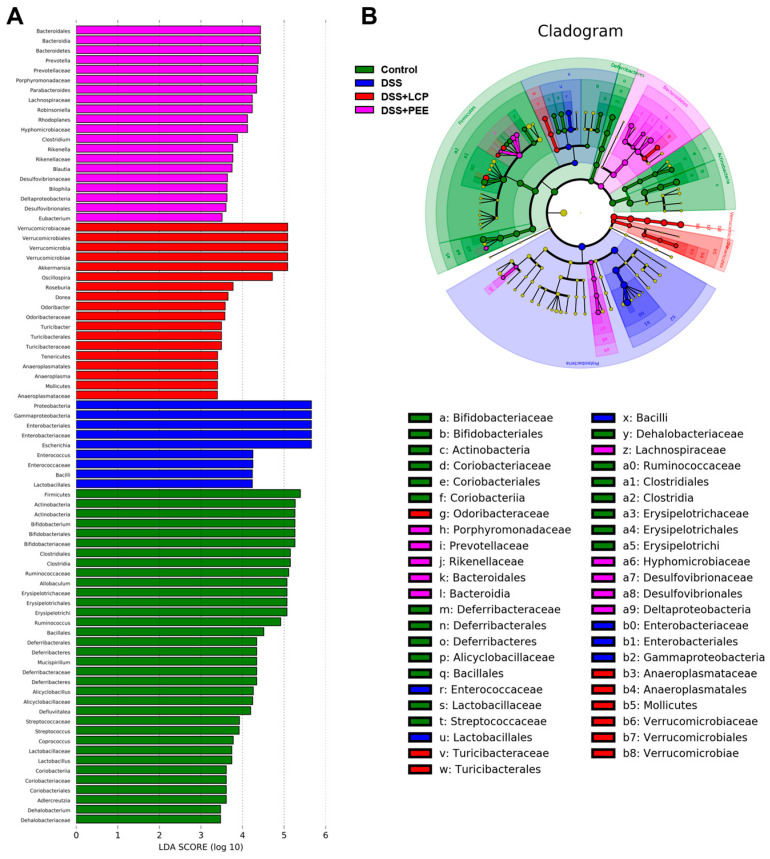
LEfSe analysis of different microbial groups. (**A**) LDA diagram. (**B**) LDA cluster diagram.

LEfSe (LDA Effect Size) analysis was used to find microbial groups with significant differences between groups and thus identify biomarkers. At the genus level, we identified that the biomarkers for the DSS group were *Bifidobacterium*, *Allobaculum*, and *Ruminococcus*, the biomarkers for the DSS group were *Escherichia* and *Enterococcus*, the biomarker for the DSS + LCP group was *Akkermansia*, and the biomarkers for the DSS + PEE group were *Prevotella* and *Parabacteroides* (Figure 7).

### 3.6. Effect of LCP and Its Fractions on Serum Metabolites in Colitis Mice

**Figure 8:**
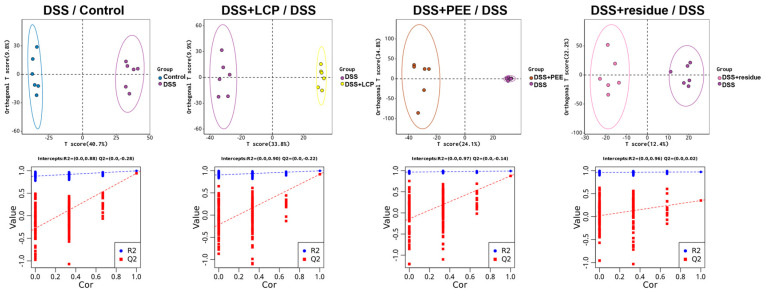
OPLS-DA scoring chart and model replacement test chart.

A total of 1528 compounds were identified by non-targeted metabolomic assay of serum using LC-MS/MS. In the PCA plots, the control and DSS groups were clearly separated, the DSS + LCP group significantly converged with the control group, and the DSS + PEE and DSS + residue groups had the most overlap with the DSS group. The relationship between metabolite expression and sample class was modeled by OPLS-DA to assess the data quality and aid in metabolic marker screening. The closer R2Y is to 1 and Q2 > 0.9 indicate the model’s good reliability and predictivity (Figure 8).

Volcano plot visualization of the differential metabolites between sample groups was screened according to three indicators, fold-change (FC) value, multiple test significance level (q-value), and VIP value for multivariate analysis, with screening criteria of FC ≥ 2 or FC ≤ 0.5, q-value < 0.05, and VIP ≥ 1. Compared with the DSS group, there were 309 metabolite expression changes in the control group, 183 metabolite expression changes in the DSS + LCP group, 30 metabolite expression changes in the DSS + PEE group, and no significantly different metabolites in the DSS + residue group. Based on the quality of the OPLS-DA model, the samples of the DSS + residue group were not subsequently analyzed.

The differential metabolites and selection of the top 10 metabolites in terms of FC value were further screened as biomarkers associated with colitis. Metabolites that were significantly improved in the DSS + LCP and DSS + PEE groups compared with the DSS group were screened according to the criteria of FC ≥ 2 or FC ≤ 0.5, q-value < 0.05, and VIP ≥ 1. No metabolites that were significantly improved in the DSS + PEE group under this screening condition were found. Compared to the control group, quinone sulfate, 3-decenoic acid, sebacic acid, prostaglandin B1, perillic acid, indoleacetic acid, and isoquinoline were significantly downregulated in the DSS group, and retinol, cortisol, andrographolide, eicosapentaenoate, isotretinoin, ibuprofen, 3-hydroxybutyric acid, and cannabidiolic acid were significantly upregulated in the DSS group, and in the DSS + LCP group, these metabolites were significantly improved toward the control group levels (Table 4).

**Figure 9:**
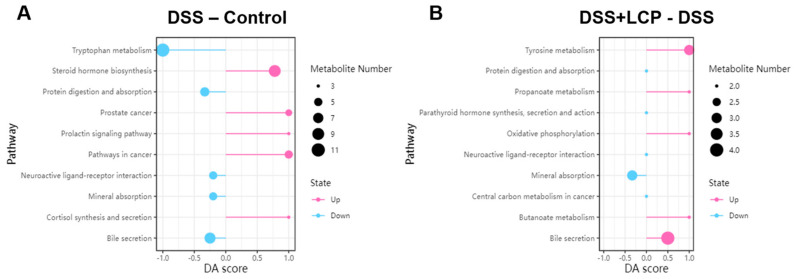
Difference abundance score chart. (**A**) DSS group compared to control group, (**B**) DSS + LCP group compared to DSS group. A DA score of 1 indicates an upregulated expression trend of all annotated differential metabolites in this pathway, and −1 indicates the opposite.

Metabolic pathway enrichment analysis of the differential metabolites based on the KEGG database was performed to reveal the metabolic pathways that underwent significant changes. Compared to the DSS group, pomace intake significantly downregulated the steroid hormone biosynthesis, prolactin signaling, Cushing’s syndrome, and linoleic acid metabolism pathways, and it upregulated the bile secretion pathway (Figure 9 and Table 5).

### 3.7. Correlation Analysis of Differential Gut Microbes and Differential Metabolites

**Figure 10:**
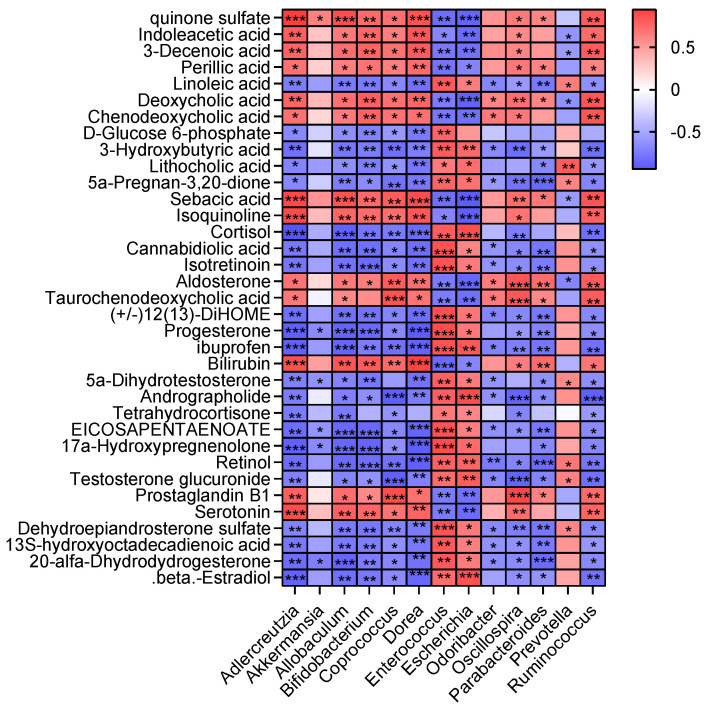
Spearman’s correlation heat map between the gut microbiota and differential metabolites. * *p* < 0.05, ** *p* < 0.01, *** *p* < 0.001.

To investigate the relationship between the gut microbiota and metabolites, Spearman’s correlation analysis was performed on 13 gut microbes with significant differences at the genus level and 35 differential metabolites (Figure 10). *Adlercreutzia*, *Allobaculum*, *Bifidobacterium*, *Coprococcus*, and *Dorea* were significantly and negatively correlated with metabolites in steroid hormone biosynthesis (D-glucose-6-phosphate, 5a-Pregnan-3,20-dione, cortisol, progesterone, 17a-hydroxypregnenolone, testosterone glucuronide, dehydroepiandrosterone sulfate, 20-alfa-dihydrodydrogesterone, and β-estradiol). The biomarker *Akkermansia* in the DSS + LCP group was significantly and negatively correlated with progesterone, 5a-dihydrotestosterone, 17a-hydroxypregnenolone, and 20-alfa-dihydrodydrogesterone in steroid hormone biosynthesis. *Adlercreutzia*, *Allobaculum*, *Coprococcus*, *Dorea*, *Oscillospira*, and *Ruminococcus* were significantly and positively correlated with metabolites of the bile secretion pathway (quinone sulfate, deoxycholic acid, chenodeoxycholic acid, taurochenodeoxycholic acid, bilirubin, and serotonin). *Adlercreutzia*, *Allobaculum*, *Bifidobacterium*, *Coprococcus*, *Dorea*, *Oscillospira*, and *Ruminococcus* were significantly and positively correlated with indoleacetic acid, 3-decenoic acid, perillic acid, sebacic acid, isoquinoline, and prostaglandin B1.

## 4. Discussion

This study found that intake of PEE and LCP had a significant preventive effect on colitis in mice, while taking fiber residue alone had no preventive effect. We speculate that the polyphenol-rich ethanol extract plays a major role in the anti-colitis effect mediated by LCP. Consistent with previous investigations, the daily intake of berries or berry pomace has been demonstrated to alleviate colitis symptoms [23,38]. In the current work, we investigated the preventive effect of the daily intake of *Lonicera caerulea* pomace and polyphenol-rich and fiber-rich ethanol extracts of LCP using a DSS-induced colitis model. We found that ingestion of *Lonicera caerulea* pomace and polyphenol-rich ethanol extract resulted in increased DAI (Figure 1B,E) and shortened colon length (Figure 1F). The clinical indicators of the *Lonicera caerulea* pomace group were closer to those of the control group, thus better preventing colitis. What is surprising is that the fiber residue group showed significant improvements in gut microbiota diversity indicators (Figure 4D), but there were no significant differences compared to the DSS group in other indicators.

MPO activity and TNF-α are markers of inflammation [39,40]. As anticipated, DSS treatment markedly elevated MPO activity compared to the control group, while dietary pomace intake notably mitigated this DSS-induced elevation [41]. No significant differences in MPO activity were observed between the groups receiving polyphenol-rich ethanol extract, fiber residue, and the DSS group (Figure 2C). The attenuation of colitis in mice by LCP might be attributed to reduced inflammatory cell infiltration, which is corroborated by pathological analyses [40,41]. Further examination of serum TNF-α levels indicated that LCP consumption significantly reduced the DSS-induced rise in TNF-α levels. The PEE group also exhibited a marked decrease in TNF-α elevation, though slightly less than in the LCP group. Conversely, the fiber residue group did not show a significant difference from the DSS group in TNF-α levels (Figure 2D). In conclusion, the LCP group demonstrated anti-inflammatory effects at both local and systemic levels, outperforming the PEE group, while the fiber residue group showed no anti-inflammatory effects.

Epithelial cells are interconnected through tight junction proteins, which regulate the permeability of the colonic barrier to resist microbial invasion. We employed immunohistochemical staining techniques to analyze the expression of tight junction proteins ZO-1, Occludin, and Claudin-1 within the colon tissues [42,43]. This analysis was pivotal in assessing and contrasting the effects of LCP and PEE on the mechanical barrier integrity of the colon [44]. When compared to the control group, the DSS group exhibited a notable diminution in the average optical density for ZO-1, Occludin, and Claudin-1, indicating a compromise in tight junction integrity. Notably, the DSS + LCP group demonstrated a significant augmentation in the average optical density for these proteins, approximating the levels observed in the control group. This suggested a restoration of tight junction protein expression. Meanwhile, the DSS + PEE group presented a significant enhancement in Claudin-1 optical density alone, with ZO-1 and Occludin only showing a non-significant increasing trend. No appreciable differences were discerned between the fiber residue group and the DSS group (Figure 3). These findings suggested that colitis may impair tight junction proteins, thus compromising the stability and structural integrity of the colon barrier. The administration of LCP and PEE appeared to modulate tight junction protein levels in mice with colitis, bolstering colon barrier stability and preserving intestinal architecture. We postulate that this modulation represents a mechanism by which LCP and PEE mitigate colitis effects, a phenomenon not observed with fiber residue intake.

The intestinal microbiota is fundamental in colitis pathogenesis [7], with dietary habits exerting a significant influence on its balance [44,45,46]. Our investigation revealed reductions in species richness and diversity within the gut microbiota of mice with colitis. The administration of LCP and PEE was observed to counteract this reduction, while fiber residue solely improved diversity (Figure 4C,E). The intake of LCP and PEE could enrich *Akkermansia* in the intestinal flora of colitis mice. *Akkermansia* proliferates in the mucus layer and feeds on mucin secreted by the host, degrading mucin and producing organic acids such as acetate and propionate and protecting the intestine from pathogens through competitive exclusion [47,48,49]. In terms of efficacy, *Akkermansia* can alleviate mucosal inflammation through the interactions between microorganisms and the host, protect intestinal barrier function, and reduce the level of inflammatory cytokines, making it a potential probiotic for improving colitis. *Akkermansia* can also increase the production of Muc2 in diabetic mice, thereby improving the thickness of the mucus layer and intestinal barrier [50]. Furthermore, *Akkermansia* has been shown to enhance the integrity of the epithelial cell layer in vitro, and its protective effect on the intestinal barrier is not entirely related to the mucus layer [51]. Combining the improved mucosal barrier and tight junction protein expression in colitis mice in the fruit residue group, it is speculated that LCP and its extract protect the colonic barrier from DSS by enriching *Akkermansia.*

Leveraging the KEGG database for metabolic pathway enrichment analysis of the differential metabolites, we discerned five pathways within the top 20 differential metabolic pathways that were significantly improved in the LCP group (Table 5). In comparison with the DSS group, fruit pomace intake markedly downregulated the steroid hormone biosynthesis, prolactin signaling, Cushing’s syndrome, and linoleic acid metabolism pathways, while the bile secretion pathway was upregulated. Given that UC patients often experience accelerated intestinal motility and abbreviated fecal reabsorption time, leading to severe diarrhea, the elevation in cortisol and its biosynthetic pathway validated the colitis-induced diarrhea model. Conversely, this pathway was significantly downregulated in the LCP group, with the formation of well-consolidated feces devoid of diarrhea, underscoring the pathway’s modulation as a potential mechanism through which LCP mitigates colitis. In IBD, compromised bile acid absorption leads to deficiencies, disrupted immune homeostasis, impaired intestinal barrier function, and IBD exacerbation [52]. The restoration of the altered bile acid secretion pathway in the LCP group signified LCP’s regulatory effect on bile acid metabolism in colitis.

Based on the screened differential metabolites, we found that cortisol participates in steroid hormone biosynthesis and was significantly elevated in the DSS group, corresponding to a significant upregulation of the steroid hormone biosynthesis pathway in the DSS group [13,17]. Patients with UC have faster intestinal peristalsis and shorter fecal reabsorption time, leading to severe diarrhea. Therefore, the upregulation of cortisol and steroid hormone biosynthesis in the DSS group confirmed the clinical response of diarrhea caused by colitis, while both were significantly downregulated in the fiber residue group, where the mice had solid feces and no diarrhea, indicating that the regulation of the steroid hormone biosynthesis pathway may be one of the important ways in which fruit residue alleviates colitis. Indole-3-propionic acid (IPA), a metabolite of the intestinal flora derived from tryptophan, plays an important role in maintaining the balance of the intestinal mucosal barrier and is significantly downregulated in the serum of colitis mice. Indoleacetic acid is a metabolite of intestinal microorganisms and an agonist of aryl hydrocarbon receptors, which are important transcription factors that can exert protective and anti-inflammatory effects in the intestine through IL-22.

While our data underscore the potential of LCP and PEE in preventing colitis and delineate the metabolic pathways of the active agents involved, the precise interactions between polyphenols, dietary fiber in LCP, and their collective effect require further exploration [53,54,55]. It has been documented that various soluble dietary fibers influence the production of phenolic metabolites during in vitro colonic fermentation [45]. Although polyphenols bound to fiber are not digested in the small intestine, microbial fermentation in the large intestine can release them [56], potentially exerting beneficial health effects. The anti-inflammatory properties of LCP may thus stem from its polyphenolic compounds, which are conveyed to the colon when bound to dietary fiber. Prospective studies may include in vitro digestion, fermentation, and cell culture assays to elucidate the activity of polyphenol complexes derived from fruit pomace and to model the in vivo digestion and metabolic processes, thereby clarifying LCP’s role in colitis amelioration [57,58]. In addition, it is possible to consider combining *Lonicera caerulea* pomace polyphenols with polysaccharides, proteins, and other substances for food applications, thereby enhancing their bioavailability and solving the problem of waste of by-product resources [59,60,61].

## 5. Conclusions

This study showed that the intake of *Lonicera caerulea* pomace (LCP) and polyphenol-enriched extract (PEE) had a positive effect on relieving colitis in mice, but that LCP was more effective than PEE, while fiber residue intake alone did not prevent the disease conditions. The effects of LCP and PEE in preventing colitis were mainly reflected in significantly slowing down the rate of weight loss, reducing the trend of the disease activity index, improving colon tissue injury and the inflammatory response, and increasing the protein expression of Claudin-1, a tight junction protein in the colon. Compared to the control group, LCP and PEE improved the species distribution of the gut microbiota. Specifically, the abundances of *Escherichia* and *Enterococcus* decreased, while the abundance of *Akkermansia* significantly increased. The intake of fiber residue enhanced the α-diversity of the gut microbiota in mice. Additionally, the intake of LCP significantly restored 15 metabolites in colitis mice and significantly improved 5 metabolic pathways. Among them, cortisol participates in steroid hormone biosynthesis. The upregulation of both in the DSS group confirmed the clinical response of diarrhea caused by colitis, which was significantly improved in the LCP group.

## Figures and Tables

**Table 1:** Experimental diet ingredients and composition.

Composition/g	Standard Diet	DSS + LCP	DSS + PEE	DSS + Residue
Casein	140.000	128.760	139.250	130.630
Corn starch	465.692	422.800	344.400	462.400
Maltodextrin	155.000	155.000	155.000	155.000
Sucrose	100.000	100.000	100.000	100.000
Soybean oil	40.000	35.200	38.400	36.200
Cellulose	50.000	11.200	49.000	17.400
Mineral	35.000	35.000	35.000	35.000
Vitamin	10.000	10.000	10.000	10.000
L-cysteine	1.800	1.800	1.800	1.800
Choline Bitartrate	2.500	2.500	2.500	2.500
TBHQ	0.008	0.008	0.008	0.008
Supplement	0.000	100.000	125.732	50.000
Total	1000.000	1002.268	1001.090	1000.938
Calorie/%				
Lipids	9.22	9.20	9.21	9.19
Protein	14.35	14.36	14.35	14.32
Carbohydrates	76.43	76.44	76.43	76.50
Total	100.00	100.00	100.00	100.00

**Table 2:** Nutritional composition of the separated components of the LCP ^a^.

Composition/(% Dry Weight)	LCP	PEE	Residue
Ash	2.35 ± 0.04	2.59 ± 0.04	1.81 ± 0.00
Lipids	4.77 ± 0.34	3.62 ± 0.04	7.63 ± 0.03
Protein	11.24 ± 0.77	1.75 ± 0.12	18.75 ± 1.26
Total dietary fiber	38.77 ± 0.20	2.27 ± 0.11	65.20 ± 0.15
Soluble dietary fiber	12.09 ± 0.80	2.27 ± 0.11	19.85 ± 0.08
Insoluble dietary fiber	26.67 ± 0.60	ND	45.34 ± 0.23
Total phenolics	9.45 ± 0.44	25.81 ± 0.11	ND

^a^ Data are the results of three parallel experiments, expressed as mean ± SD, ND means not detected.

**Table 3:** Contents of polyphenol chemical substances.

No.	Name	RT/min	Molecular Formula	Transition	Linearity (r^2^)	Dynamic Range (ng/mL)	Quantification/ (mg/100 g LCP)
1	Astragalin	4.91	C_21_H_20_O_11_	447.1–255.0	0.99270	2–500	1.32 ± 0.03
2	Isochlorogenic acid C	4.92	C_25_H_24_O_12_	515.0–353.0	0.99017	2–500	46.95 ± 0.94
3	Naringenin	6.10	C_15_H_12_O_5_	271.0–151.0	0.99748	2–500	0.81 ± 0.02
4	Daidzin	4.25	C_21_H_20_O_9_	415.0–252.0	0.99919	2–500	0.00 ± 0.00
5	Neodiosmin	4.97	C_28_H_32_O_15_	607.0–299.0	0.99880	2–500	2.02 ± 0.04
6	Cyanidin-3-O-galactoside	3.80	C_21_H_21_ClO_11_	447.0–284.0	0.99984	2–500	231.35 ± 2.31
7	Aromadendrin	5.28	C_15_H_12_O_6_	287.0–125.0	0.99791	2–500	0.57 ± 0.01
8	Amentoflavone	6.35	C_30_H_18_O_10_	537.0–375.0	0.99783	2–500	0.78 ± 0.02
9	1,5-Dicaffeoylquinic acid	4.25	C_25_H_24_O_12_	515.0–353.0	0.99533	2–500	0.01 ± 0.00
10	7-Hydroxycoumarin	4.91	C_9_H_6_O_3_	161.0–133.0	0.99419	2–500	0.48 ± 0.01
11	Procyanidin B2	4.04	C_30_H_26_O_12_	577.0–289.0	0.99947	2–500	0.57 ± 0.01
12	Cyanidin-3-O-glucoside	3.78	C_21_H_21_ClO_11_	447.0–147.0	0.99867	2–500	388.31 ± 8.93
13	Epicatechin	4.22	C_15_H_14_O_6_	289.0–109.0	0.99727	2–500	1.07 ± 0.02
14	Catechin	3.94	C_15_H_14_O_6_	289.0–109.0	0.99604	2–500	21.62 ± 0.43
15	Chlorogenic acid	3.92	C_16_H_18_O_9_	353.0–191.0	0.99489	2–500	55.22 ± 1.10
16	Quercetin	5.65	C_15_H_10_O_7_	301.0–151.0	0.99962	2–500	19.60 ± 0.39
17	Caffeic acid	4.18	C_9_H_8_O_4_	179.0–135.0	0.99818	2–500	3.23 ± 0.06
18	Phlorizin	5.19	C_21_H_24_O_10_	435.0–273.0	0.99930	2–500	4.26 ± 0.09
19	Phloretin	6.04	C_15_H_14_O_5_	273.0–123.0	0.99878	2–500	0.10 ± 0.00

**Table 4:** Metabolites significantly improved in the DSS + LCP group.

No	Name	DSS/Control		DSS + LCP/DSS	
		Trend	FC	Trend	FC
1	quinone sulfate	↓ **	0.0706	↑ **	191.0376
2	3-Decenoic acid	↓ **	0.2587	↑ **	2.5648
3	Sebacic acid	↓ ***	0.1405	↑ **	4.1414
4	Prostaglandin B1	↓ **	0.3287	↑ *	2.5800
5	Perillic acid	↓ ***	0.3302	↑ *	1.6749
6	Indoleacetic acid	↓ **	0.3949	↑ *	2.2045
7	Isoquinoline	↓ **	0.3638	↑ *	1.8321
8	Retinol	↑ ***	4.9682	↓ ***	0.2622
9	Cortisol	↑ **	6.8051	↓ **	0.1572
10	Andrographolide	↑ **	21.5829	↓ **	0.0643
11	Eicosapentaenoate	↑ ***	8.6958	↓ **	0.2499
12	Isotretinoin	↑ **	4.9295	↓ *	0.2666
13	ibuprofen	↑ **	2.2656	↓ *	0.5363
14	3-Hydroxybutyric acid	↑ ***	2.0353	↓ *	0.6467
15	Cannabidiolic acid	↑ **	4.7801	↓ *	0.3106

* *p* < 0.05, ** *p* < 0.01, *** *p* < 0.001, ↑indicates upregulation of the pathway, ↓indicates downregulation.

**Table 5:** Significantly improved metabolic pathways in the DSS + LCP group.

No	Pathway	DSS/Control		DSS + LCP/DSS		Pathway ID
		Trend	DA Scorea	Trend	DA Scorea	
1	Steroid hormone biosynthesis	↑***	0.78	↓ *	−1.00	map00140
2	Bile secretion	↓ ***	−0.25	↑ *	1.00	map04976
3	Prolactin signaling	↑ ***	1.00	↓ ***	−0.33	map04917
4	Cushing’s syndrome	↑ ***	1.00	↓ ***	0.00	map04934
5	Linoleic acid metabolism	↑ **	1.00	↓ *	0.00	map00591

DA score of 1 indicates an upregulated expression trend of all annotated differential metabolites in this pathway, and −1 indicates the opposite. * *p* < 0.05, ** *p* < 0.01, *** *p* < 0.001, ↑indicates upregulation of the pathway, ↓indicates downregulation.

## Data Availability

The original contributions presented in this study are included in the article’s material. Further inquiries can be directed to the corresponding author.
